# Exploring the current state of clinical and practical teaching in obstetrics and gynecology in the era of competency-based education: a nationwide survey among German teaching coordinators

**DOI:** 10.1186/s12909-024-05138-2

**Published:** 2024-02-21

**Authors:** Bastian Meyer, Fabian Riedel, Niklas Amann, Anna Graf, Antonia Stuehrenberg, Viktoria Ritter, Markus Wallwiener, Sabine Heublein, Florian Recker, Martin Weiss, Maximilian Riedel

**Affiliations:** 1 Department of Gynecology and Obstetrics, Klinikum Rechts Der Isar, Technical University Munich, Munich, Germany; 2 Department of Gynecology and Obstetrics, Heidelberg University Hospital, Heidelberg, Germany; 3 Department of Gynecology and Obstetrics, Friedrich–Alexander-University Erlangen–Nuremberg (FAU), Erlangen, Germany; 4 Department of Gynecology and Obstetrics, Halle University, Halle, Germany; 5 Department of Gynecology and Obstetrics, Bonn University Hospital, Bonn, Germany; 6 Department of Women’s Health, University of Tübingen, Tübingen, Germany; 7 NMI Natural and Medical Sciences Institute, University of Tübingen, Reutlingen, Germany

**Keywords:** Medical education, Teaching coordinators, Curriculum development, German universities, Medical curriculum, Competency-based education, NKLM

## Abstract

**Background:**

Obstetrics and gynecology (OB/GYN) is an essential medical field that focuses on women’s health. Universities aim to provide high-quality healthcare services to women through comprehensive education of medical students. In Germany, medical education is undergoing a phase of restructuring towards the implementation of competency-based learning. The objective of the current survey was to gain insights into the teaching methods, resources, and challenges at German medical universities in the field OB/GYN. This aims to document the current state of medical education and derive potential suggestions for improvements in the era of competency-based learning. The survey was conducted with teaching coordinators from the majority of OB/GYN departments at German universities.

**Methods:**

A questionnaire was sent to the teaching coordinators in all 41 OB/GYN departments at German university hospitals. The survey was delivered via email with a link to an online survey platform.

**Results:**

The study received 30 responses from 41 universities. Differences were observed in the work environment of teaching coordinators concerning release from clinical duties for teaching purposes and specialized academic training. Overall, medical education and student motivation were perceived positively, with noticeable gaps, particularly in practical gynecological training. Deficiencies in supervision and feedback mechanisms were also evident. Subfields such as urogynecology and reproductive medicine appear to be underrepresented in the curriculum, correlating with poorer student performance. E-learning was widely utilized and considered advantageous.

**Conclusion:**

The present study provides valuable insights into the current state of medical education in OB/GYN at German universities from the perspective of teaching experts. We highlight current deficits, discuss approaches to overcome present obstacles, and provide suggestions for improvement.

**Supplementary Information:**

The online version contains supplementary material available at 10.1186/s12909-024-05138-2.

## Background

Obstetrics and gynecology (OB/GYN) is an essential medical field that focuses on women’s health. Providing medical students with comprehensive and up-to-date education in this area is vital to ensuring the delivery of high-quality healthcare services to women. In Germany, undergraduate medical studies are divided into three phases and typically take six years in total to complete. The initial two pre-clinical years focus more on fundamental sciences, such as biology, physics, anatomy, physiology, and (bio-)chemistry, and are followed by three years of comprehensive clinical education. The clinical curriculum encompasses all major and minor specialties, which are taught through lectures, seminars, and bedside teaching [1]. The final year – called the “Praktisches Jahr” (PJ) – involves full-time training in usually three 16-week blocks that consist of internal medicine, (general) surgery, and an elective specialty. The final year allows students to gain hands-on experience in different medical settings, such as hospitals, clinics, and other healthcare facilities [2].

In the German medical education system, only university hospitals and their affiliated teaching hospitals are authorized to provide medical teaching, examinations, and licensing. Teaching coordinators – who hold an official position within a department (such as OB/GYN) – are appointed by the medical faculty and are usually senior physicians who work part-time in this role. Typically, one person with expertise and experience in medical teaching is responsible for the position in each medical department. Teaching coordinators are responsible for organizing and supervising all theoretical and practical student courses. They also develop the curriculum and implement innovative teaching formats within their respective departments. Moreover, along with other teaching coordinators from various departments, these coordinators often serve as members of the central decision-making body when it comes to coordinating medical teaching. Teaching coordinators usually also participate in teaching during lectures, seminars, and hands-on courses together with the teaching staff [3].

Medical education is monitored and evaluated through a comprehensive and rigorous system to ensure high standards of teaching and learning at German university hospitals. The process is governed by a combination of federal regulations and state-specific laws, as well as, university-specific policies. The overarching legal framework for medical education in Germany is established by the Medical Licensing Regulations for Physicians (ÄAppO), set forth by the Ministry of Health [4]. This framework not only governs the admission process into the medical profession, but it also outlines the guidelines for medical study content. The Joint Federal Committee (G-BA), which includes physicians, dentists, psychotherapists, hospitals, and health insurance funds, functions as the primary decision-making authority in this regard [5]. The G-BA is empowered to issue educational guidelines that universities must integrate into their curricula. While individual medical faculties at universities enjoy a certain level of autonomy in designing their curricula, they are bound to comply with these legal stipulations. Additionally, internal quality management systems within universities regularly assess teaching effectiveness, student satisfaction, and learning outcomes. Feedback from students and faculty is a crucial component of this evaluation process [6]. Professional societies and specialized committees, such as the German Society of Gynecology and Obstetrics (DGGG) and its subdivision, the “Young Forum” — a group representing young physicians in training — play a significant but indirect role in shaping undergraduate and postgraduate medical education [7]. These organizations foster an environment of exchange and networking among medical professionals and trainees, offering a platform for the discussion of innovative educational methods and contributing to the advancement of OB/GYN education, although, without directly influencing the curriculum.

In recent years, medical education in Germany has undergone important developments and challenges. First, the disruption to medical education caused by the COVID-19 pandemic may have accelerated the advancement of online teaching and learning, as suggested by studies both in Germany [8–10] and globally [11–13]. Second, Germany is currently in the process of implementing the “National Competency-Based Learning Objectives for Undergraduate Medical Education” (NKLM) as part of the Masterplan for Medical Studies 2020 with the aim of improving – and setting new – quality standards in medical education [14, 15]. The Masterplan for Medical Studies 2020 sets out a number of key objectives in medical education, including the goal of strengthening the practical skills and clinical competencies of medical students, promoting interdisciplinary training, increasing digitalization in medical education and patient-oriented care. This approach no longer mandates a rigid division between clinical and preclinical content, thus, allowing for a more integrated approach to medical education [16]. Some of the specific measures proposed by the Masterplan 2020 include expanding practical training opportunities for medical students, promoting the use of e-learning and other digital tools in medical education, increasing the number of teaching staff and more exchange programs for medical students. [13, 14].

The NKLM serves as a comprehensive framework defining the expected learning outcomes for undergraduate medical education in German. It is structured around various competency domains in medical practice, including medical expertise and patient communication [17]. Within each domain, the NKLM specifies a detailed set of learning objectives that medical students are expected to achieve during their undergraduate studies. These objectives aim to establish a clear and quantifiable standard for medical education, ensuring that graduates possess the essential knowledge, skills, and attitudes required for safe and effective patient care. The NKLM outlines four distinct "competence depths" which include cognitive, affective, and action-oriented competencies. A major challenge in developing curricula is to efficiently match the teaching content with the corresponding depth of competency, particularly in the context of the limited time allocated for each topic. [16]. The NKLM is not supposed to be a curriculum in itself; rather, it is a set of learning objectives that can be incorporated into existing curricula. Moreover, it is intended to provide guidance to medical schools and faculty members when it comes to developing and implementing curricula that meet the needs of modern medical practice [11]. The NKLM will become a mandatory part of the medical licensing regulations in the future. The NKLM catalogue has been and is currently further developed and evaluated in collaboration with the medical faculties [18].

The implementation of competency-based medical education represents a paradigm shift in German medical education and has also attracted renewed interest worldwide and in various medical settings in recent years among educators [19–22]. Therefore, analyzing the current state of medical education during this transformation holds considerable potential. This is particularly pertinent in the field of OB/GYN, where the nature of the discipline inherently requires a strong emphasis on practical and manual skills. To that end, in cooperation with the Young Forum of the German Society for Gynecology and Obstetrics, we conducted a nationwide survey among German teaching coordinators with the goal of providing a comprehensive report of the current state of medical education in OB/GYN from their perspective with a focus on the developments with regard to competency-based education and practical training. To the best of our knowledge, this is the first survey specifically addressing the teaching coordinators as highly relevant stakeholder in the medical education system in Germany. Given the transformative nature of the NKLM and the global trend towards competency-based learning, we seek to offer insights that extend beyond national boundaries. In this context, we primarily address three inquiries:

I) How does the structure of medical education in OB/GYN differ among universities in Germany, and what level of importance is given to practical training in this field?
II) What kind of challenges are teaching coordinators facing in managing their teaching responsibilities alongside their clinical work, and how do they assess the current state of teaching and education at their OB/GYN institution?
III) What approaches and solutions can be developed to effectively address these challenges and to improve medical education in OB/GYN in the best interest of the stakeholders involved?

## Methods

### Data acquisition and survey design

The authors of the present study created a questionnaire that was utilized for the first time in this survey. The questionnaire was disseminated among teaching coordinators at all 41 departments of OB/GYN at both public (*n* = 37) and private (*n* = 4) university hospitals in Germany via an email invitation. If the name of the teaching coordinator was available online or was otherwise known, the email was directly addressed to this individual (*n* = 24). However, if the responsible person could not be located, the email was directed to the secretary’s office of the respective department or clinic with a request to forward it (*n* = 17). The email explained the aim of the study and included a weblink to the online survey hosted on *SurveyMonkey* (Survey Monkey Inc., San Mateo, CA, USA).

Participation in the study was entirely voluntary, and all collected data were anonymized. Informed consent was obtained electronically before the questionnaire was administered, and each participant agreed to data analysis electronically. Responses were collected over a period of 49 days between 19 April and 7 June 2021. After four weeks, a survey reminder was sent once to all departments via email. No compensation for participation was provided. Answers were collected anonymously, and if more than one teaching coordinator was present in a department, the email was sent to each coordinator. A rough regional allocation of responses was possible using respondents’ postal code.

The questionnaire that was used in the survey consisted of 116 items, 68 of which were either dichotomous or classification questions and 48 of which used either a numeric rating scale or five-point Likert scale ratings, in which participants indicated their level of agreement or disagreement with a given statement (1 = *strongly disagree (–)*, 2 = *disagree (-)*, 3 = *neither agree nor disagree (-/* +*)*, 4 = *agree (* +*)*, 5 = *strongly agree (*+ +*)*).

The original questionnaire was divided into two thematic blocks. The first part of the survey addressed the current state of affairs as well as future developments and challenges in teaching OB/GYN. The second part was implemented in order to capture experiences during the COVID-19 pandemic and specifically focused on the influences that the pandemic had had on teaching, the shift to online learning, and digitization in medical education in general. Due to the volume of the data and the lack of thematic overlap, the results of the COVID-19-specific second part of the survey were published separately in 2022 [3].

### Statistical analysis

Statistical assessment was conducted via *Microsoft Excel* (Microsoft, Version 2021). Tables and figures were generated in *Microsoft Word* and *Microsoft PowerPoint* (Version 2022, Microsoft Corp., Redmond, WA, USA). Mean and relative values were calculated descriptively for each individual item.

## Results

### Demographic characteristics and work experiences of the surveyed teaching coordinators

A total of 41 university hospitals and their corresponding teaching coordinators were included in this study. Of these, 73% (*n* = 30) participated and completed the questionnaire. The sources of the responses were distributed among all regions of Germany, as indicated by the regional distribution of the postal codes (Supp. Tab. 1). On average, the teaching coordinators were 38 years old (standard deviation: 7.5 years); 57% (*n* = 17) were male, while 43% (*n *= 13) were female. A majority of the respondents (57%; *n* = 17) had more than four years of experience as teaching coordinators. In terms of their clinical positions, 50% (*n* = 15) held leading physician positions (“Chefarzt” or “Oberarzt”), 23% (*n* = 7) had completed their specialist training (“Facharzt”), and another 23% (*n* = 7) were undergoing specialist training (“Assistenzarzt”) in OB/GYN.

### Working environment of the OB/GYN lecturers

Two-thirds (65%; *n* = 22) of the university lecturers in OB/GYN stated that they are released from their clinical duties for medical education. Regular student evaluations of medical teaching and courses are conducted in all departments (100%; *n* = 29). The survey indicated that it is mandatory for students at 34% (*n* = 10) of departments to evaluate finished courses, for example, in order to receive course credit. Moreover, 72% (*n* = 21) of the OB/GYN departments provide direct and individual feedback to each lecturer about the lecturer’s evaluation results. Didactic training is mandatory for lecturers at 41% (*n* = 12) of universities, and 48% (*n* = 14) of departments regularly recruit external lecturers. A majority of university hospitals (55%; *n* = 16) recognize and reward good teaching (e.g., via an annual honor or prize), while 62% (*n* = 18) allocate (financial) resources to clinical departments based on these departments’ performance in student evaluations.

### Methods of course performance assessment in OB/GYN departments

Most departments use multiple-choice tests (100%; *n* = 29), oral examinations (52%; *n* = 15), and – to a lesser extent – open-ended text questions (14%; *n* = 4) for student examinations. Additional teaching formats – such as the Objective Structured Clinical Examination (OSCE) or Mini-Clinical Evaluation Exercises (Mini-CEX) – are used by 55% (*n* = 16) and 24% (*n* = 7) of OB/GYN departments, respectively, for conducting performance assessments.

### Implementation of theoretical training in OB/GYN departments

Theoretical teaching in OB/GYN primarily consists of classical lectures (100%; *n* = 30) and seminars (93%; *n* = 27). Problem-based learning (POL) is less commonly used and is offered at 28% (*n* = 8) of OB/GYN departments. E-learning in the form of self-established or recorded formats (e.g., digital access to recorded lectures and seminars) is implemented at 76% (*n* = 22) of universities, while 93% (*n* = 27) of medical faculties provide free access to commercial digital learning platforms, such as *Amboss* or *Thieme online*.

Attendance at lectures is usually non-compulsory (93%; *n* = 27), whereas it is mostly mandatory for seminars (93%; *n* = 26). Only 34% (*n* = 10) of OB/GYN departments allow students to prioritize and focus on special subjects of interest. Voluntary, non-curricular teaching offerings (e.g., pre-exam review courses) are organized by 69% (*n* = 20) of OB/GYN departments. Cross-curricular courses that incorporate students from different degree programs (e.g., midwifery or nursing) are less commonly available (28%; *n* = 8). Mentoring programs for students with high levels of interest in a later OB/GYN career are offered at 34% (*n* = 10) of the universities.

### Evaluation of clinical examination and practical skills training in OB/GYN

A high proportion (79%; *n* = 23) of the teaching coordinators stated that their institutions have established standards and guidelines for practical training in OB/GYN clinical examination techniques. Bedside teaching during clinical clerkships (“Blockpraktika”) (97%; *n* = 28) and clinical simulation centers (“skills labs”) (86%; *n* = 25) are widely used for practical training. The standard gynecological examination (93%; *n* = 26), the examination of the female breast (100%; *n* = 28), and basic birth mechanics (96%; *n* = 27) are typically taught using clinical phantom models.

Most teaching coordinators agree that the presence of medical students during the gynecological examination of a female patient by a physician (82%; *n* = 23), during the clinical examination of the female breast (75%; *n* = 21), and during a vaginal delivery (64%; *n* = 18) is a crucial learning objective. By contrast, the teaching coordinators consider it less important for medical students to conduct these examinations personally. Specifically, 29% of the teaching coordinators (*n* = 8) stated that they regard the gynecological examination as a notable learning objective, while 32% (*n* = 9) stated that they view the breast examination as an important aspect of their instruction. While a significant number of respondents (98%; *n* = 28) agreed that all medical students should have a theoretical understanding of the gynecological examination, only a small fraction (39%; *n* = 11) stated that they believe that it is necessary for students to gain practical mastery of the procedure. Most of the teaching coordinators agreed that practical examination skills should be learned during elective clinical clerkships (“Famulatur”) or during the final-year OB/GYN rotation (“Praktisches Jahr”) (weighted average Likert scale: 4.46) rather than during compulsory clinical clerkships (“Blockpraktika”) (weighted average Likert scale: 3.43). The majority of teaching coordinators (68%; *n* = 19) stated that they consider it acceptable to perform a vaginal examination while the patient is under anesthesia before a planned surgery as long as the patient’s prior consent has been obtained.

Only a small number of OB/GYN departments use medical training aids such as transabdominal (25%; *n* = 7) or transvaginal (21%; *n* = 6) ultrasound trainers, hysteroscopy trainers (33%; *n* = 9), and laparoscopy trainers (57%; *n* = 16). Additionally, less than half of the surveyed gynecological departments provide theoretical or practical instruction on suturing techniques (46%; *n* = 13) (Table 1).

**Table 1:** Respond if the following statements regarding teaching with trainers / phantoms / models apply to your clinic

	**yes**		**no**		**n/a**		**total**
	**%**	***n*** ** = **	**%**	***n*** ** = **	**%**	***n*** ** = **	***n*** ** = **
The (vaginal) gynecological examination is taught using a phantom / model	93	26	7	2	0	0	28
The examination of the female breast is taught using a phantom / model	100	28	0	0	0	0	28
The mechanics of childbirth are taught using a classical pelvic phantom	96	27	4	1	0	0	28
The mechanics of childbirth are taught using a birthing simulator or birthing doll	68	19	29	8	4	1	28
A transabdominal ultrasound simulator for obstetrical questions (e.g., fetal biometry) is available	32	9	61	17	7	2	28
A transabdominal ultrasound simulator for obstetrical questions (e.g., fetal biometry) is used in student education	25	7	68	19	7	2	28
A transvaginal ultrasound simulator is available in the clinic	21	6	75	21	4	1	28
A transvaginal ultrasound simulator is used in student education	21	6	75	21	4	1	28
A laparoscopy trainer is available in the clinic	82	23	18	5	0	0	28
A laparoscopy trainer is used in student education	57	16	36	10	7	2	28
A hysteroscopy trainer is available in the clinic	39	11	57	16	4	1	28
A hysteroscopy trainer is used in student education	32	9	64	18	4	1	28
Suturing techniques are taught theoretically	46	13	46	13	7	2	28
Suturing techniques are practiced on a phantom / model	46	13	46	13	7	2	28

At 38% (*n* = 11) of the OB/GYN departments, communication training and simulated patient scenarios are provided for certain gynecological or obstetric issues, for example, when delivering the news of a tumor diagnosis.

### Opportunities, motivation, and challenges of final-year medical students in OB/GYN

Seventy-five percent (*n* = 21) of the surveyed gynecological departments provide an explicit catalog of learning objectives for the final year, which is tracked in a logbook. Most students (82%; *n* = 23) have the opportunity to specialize in certain fields of interest during their rotation. In 82% (*n* = 23) of the departments, final-year students are granted access to the clinic’s internal digital patient management system, such as *SAP* or *Orbis*.

The teaching coordinators hold a positive view of their final-year students’ motivation and commitment (weighted average Likert scale: 4.25), and they acknowledge the students’ sense of responsibility toward their tasks in regular patient care (weighted average Likert scale: 4.32). From the perspective of the lecturers, good and dedicated teaching is highly valued by students (weighted Likert scale: 4.00). A large majority of the teaching coordinators (87%; *n* = 27) recognize their final-year students’ contribution to the smooth running and functioning of the clinic. The teaching coordinators hold the view that final-year experiences are crucial for future specialist training (weighted average Likert scale: 4.36) and that good clinical teaching and medical education are therefore essential (weighted average Likert scale: 4.58). By contrast, only 40% of the teaching coordinators agree that students are adequately prepared for day-to-day clinical life after the completion of the final year. Only a minority of respondents agree with the statement that the time allocated for teaching final-year students in clinical routine is adequate (28%; *n* = 8) and stated that the staff and financial resources required for optimal teaching are sufficient (35%; *n* = 9) (Table 2).

**Table 2:** Respond if the following statements regarding the final year (“Praktisches Jahr”) apply to your clinic

	**completely disagree (1)**		**disagree (2)**		**neither agree nor disagree (3)**		**agree (4)**		**completely agree (5)**		**n/a**		**total**	**weighted average**
	**%**	***n*** ** = **	**%**	***n*** ** = **	**%**	***n*** ** = **	**%**	***n*** ** = **	**%**	***n*** ** = **	**%**	***n*** ** = **	***n*** ** = **	
Most students are highly motivated and engaged	0	0	0	0	4	1	68	19	29	8	0	0	28	4.25
Most students handle the tasks assigned to them very responsibly	0	0	0	0	0	0	68	19	32	9	0	0	28	4.32
The contributions of final-year students are adequately appreciated	4	1	21	6	25	7	43	12	7	2	0	0	28	3.29
Final-year students play a crucial role in the smooth functioning of the clinic	0	0	4	1	7	2	46	13	39	11	4	1	28	4.26
There is enough time allocated for teaching final-year students in the clinical routine	7	2	21	6	25	7	43	12	0	0	4	1	28	3.07
The personnel and financial resources are sufficient to ensure optimal teaching	19	5	38	10	8	2	31	8	4	1	0	0	26	2.62
Priority is given to completing routine clinical tasks (e.g., blood collection) before teaching	0	0	11	3	46	13	21	6	21	6	0	0	28	3.54
The final year is crucial for choosing specialty training after graduation	0	0	4	1	0	0	54	15	43	12	0	0	28	4.36
Having completed the final year in one’s own clinic is a prerequisite for a possible application as a resident physician	0	0	0	0	14	4	50	14	36	10	0	0	28	4.21
Upon completion of their final year, students are optimally prepared for the clinical environment	4	1	11	3	46	13	36	10	4	1	0	0	28	3.25
I wish that my own final-year rotation in OB/GYN had been like the one at my current clinic	4	1	7	2	43	12	25	7	11	3	11	3	28	3.36

### Evaluating student success and teaching in OB/GYN

Students’ overall success in learning during their clinical training is rated the highest in the areas of gynecological history-taking (mean school grades ranging from “1” (very good) to “6” (unsatisfactory): 2.00) and obtaining a general overview of the gynecological profession (average school grade: 2.11). The learning success of obstetrics (mean school grade: 2.23) and operative gynecology (mean school grade: 2.5) are also well-regarded. Conversely, learning success in the subfields of endocrinology and reproductive medicine (mean school grade: 3.64) and urogynecology (mean school grade: 3.60) are rated the lowest (Table 3). More than half (54%; *n* = 14) of the respondents indicated their wish for the focus in medical teaching to shift from theoretical training in the form of lectures toward more practical exercises with patient interaction and interactive seminars (weighted Likert scale: 3.65).

**Table 3:** Evaluate students’ learning outcomes (in school grades ranging from excellent (“1”) to unsatisfactory (“6”)) in the corresponding sub-specializations and subject areas in OB/GYN teaching

	excellent (1)		good (2)		satisfactory (3)		adequate (4)		poor (5)		unsatisfactory (6)		n/a		total	weighted average
	%	*n* =	%	*n* =	%	*n* =	%	*n* =	%	*n* =	%	*n* =	%	*n* =	*n* =	
Overview of the gynecological professional field (hospital and private practice)	21	6	57	16	11	3	11	3	0	0	0	0	0	0	28	2.11
gynecological medical history	32	9	46	13	14	4	4	1	4	1	0	0	0	0	28	2
practical gynecological examination techniques	11	3	36	10	29	8	11	3	7	2	7	2	0	0	28	2.89
operative gynecology	18	5	32	9	39	11	7	2	0	0	4	1	0	0	28	2.5
senology (study of breast)	11	3	39	11	25	7	18	5	4	1	0	0	4	1	28	2.63
conservative gynecological oncology	7	2	36	10	29	8	14	4	7	2	4	1	4	1	28	2.89
obstetrics and pre-natal medicine	18	5	46	13	18	5	11	3	0	0	0	0	7	2	28	2.23
gynecological emergencies	14	4	29	8	32	9	18	5	4	1	0	0	4	1	28	2.67
obstetric emergencies	18	5	25	7	25	7	21	6	4	1	0	0	7	2	28	2.65
interdisciplinary emergencies	4	1	32	9	21	6	29	8	4	1	4	1	7	2	28	3.08
endocrinology / reproductive medicine	4	1	18	5	25	7	14	4	18	5	11	3	11	3	28	3.64
urogynecology	4	1	21	6	18	5	18	5	21	6	7	2	11	3	28	3.6

The time available for teaching was deemed adequate for most of the sub-specializations in OB/GYN (weighted Likert scale for all sub-specializations ranging from “1” (significantly too little time) to “5” (significantly too much time): 2.61). However, the teaching coordinators raised concern as to whether there is insufficient time to teach endocrinology and reproductive medicine, urogynecology, and the practical gynecological examination (Table 4).

**Table 4:** Respond regarding how you evaluate the available time (ranging from significantly too little to significantly too much) for the corresponding sub-specializations and subject areas in OB/GYN teaching

	significantly too little		somewhat too little		adequate		somewhat too much		significantly too much		n/a		total
	%	*n* =	%	*n* =	%	*n* =	%	*n* =	%	*n* =	%	*n* =	
gynecological history	4	1	12	3	81	21	4	1	0	0	0	0	26
practical gynecological examination techniques	8	2	52	13	40	10	0	0	0	0	0	0	25
operative gynecology	8	2	27	7	54	14	12	3	0	0	0	0	26
conservative gynecological oncology	12	3	35	9	54	14	0	0	0	0	0	0	26
obstetrics and pre-natal medicine	0	0	12	3	88	23	0	0	0	0	0	0	26
endocrinology and reproductive medicine	12	3	50	13	31	8	4	1	4	1	0	0	26
senology	4	1	15	4	77	20	4	1	0	0	0	0	26
urogynecology	15	4	38	10	46	12	0	0	0	0	0	0	26
obstetrical emergencies	4	1	23	6	73	19	0	0	0	0	0	0	26
gynecological emergencies	4	1	19	5	77	20	0	0	0	0	0	0	26

**Fig. 1:**
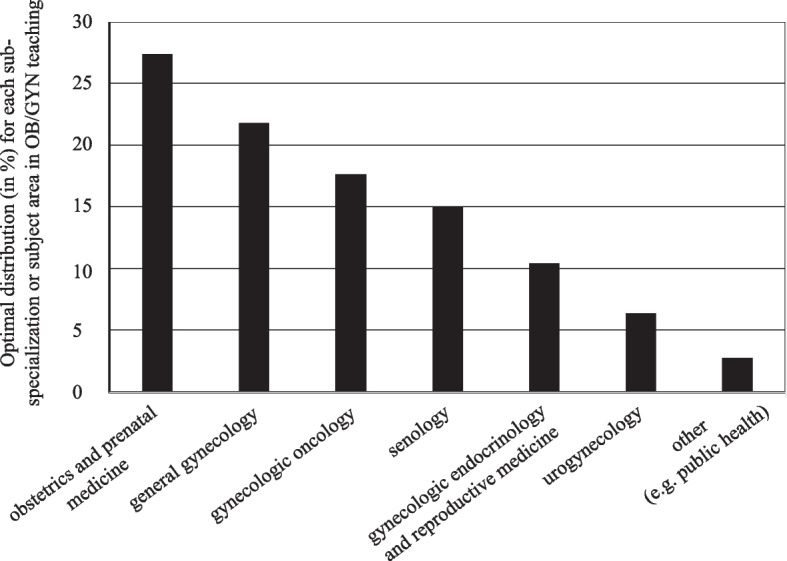
Respond regarding what the optimal distribution (in %) for each sub-specialization or subject area in OB/GYN teaching should be

When asked what the optimal distribution of time for each sub-specialization should be, the teaching coordinators rated general obstetrics and pre-natal medicine the highest (27%), followed by general gynecology (22%) (Fig. 1).

The use of digital learning platforms was widely regarded to be advantageous for student education (weighted Likert scale: 4.21). The teaching coordinators stated that they believe that e-learning can enhance students’ theoretical abilities (weighted Likert scale: 3.96) but that it may not significantly improve students’ practical abilities (weighted Likert scale: 2.71). The teaching coordinators do not support the view that compulsory attendance leads to improved teaching quality (weighted Likert scale: 2.42), and they also disagree with the notion that e-learning platforms can serve as a substitute for seminars (weighted Likert scale: 2.11) or practical (clinical) training (weighted Likert scale: 1.57). The time-saving benefits of e-learning were generally viewed as controversial (weighted Likert scale: 3.67). The teaching coordinators were divided in their opinion as to whether e-learning can improve exam performance (weighted Likert scale: 3.38) (Table 5).

**Table 5:** Respond regarding the extent to which you agree or disagree with the following statements about digitalization and e-learning in medical education

	completely disagree (1)		disagree (2)		neither agree nor disagree (3)		agree (4)		completely agree (5)		n/a		total	weighted average
	%	*n* =	%	*n* =	%	*n* =	%	*n* =	%	*n* =	%	*n* =	*n* =	
The use of digital learning platforms has a beneficial impact on student education	0	0	7	2	7	2	43	12	43	12	0	0	28	4.21
The use of digital learning platforms improves students’ theoretical abilities	4	1	7	2	7	2	54	15	29	8	0	0	28	3.96
The use of digital learning platforms improves students’ practical abilities	11	3	32	9	36	10	18	5	4	1	0	0	28	2.71
The use of digital learning platforms can replace lectures in student education	14	4	36	10	18	5	21	6	11	3	0	0	28	2.79
The use of digital learning platforms can replace seminars in student education	21	6	54	15	18	5	7	2	0	0	0	0	28	2.11
The use of digital learning platforms can replace clinical internships (“Blockpraktika”) in student education	54	15	39	11	4	1	4	1	0	0	0	0	28	1.57
The use of digital learning platforms leads to better exam results in the state examination	4	1	0	0	54	15	29	8	7	2	7	2	28	3.38
Using digital learning platforms for the pure transfer of knowledge allows more time for the practical application of what has been learned in clinical training	4	1	4	1	25	7	54	15	11	3	4	1	28	3.67
Stricter attendance is a means of improving the quality of teaching	12	3	50	13	27	7	8	2	4	1	0	0	26	2.42

### General assessments and observations of student teaching in OB/GYN

Medical teaching is generally viewed positively by teaching coordinators. A majority of these coordinators indicated a high level of appreciation for the current organization of student teaching in their department and stated that they would have welcomed such teaching during their own student days (weighted Likert scale: 3.48). Furthermore, the teaching coordinators reported that medical students are highly motivated (weighted Likert scale: 4.21) and exhibit a strong appreciation for good teaching (weighted Likert scale: 4.00). However, the teaching coordinators widely perceived student teaching in clinical practice as more of a burden than a source of enrichment (weighted Likert scale: 3.36).

With regard to the gender-specific interest of medical students in OB/GYN, the teaching coordinators expressed mixed opinions as to whether the low interest of male students in the specialty is problematic (weighted Likert scale: 2.76), and they rejected the notion that offering specialized teaching to male students would increase these students’ interest in this area (weighted Likert scale: 2.46). The teaching coordinators presented a mixed perspective regarding whether student teaching adequately takes the significant surgical component of OB/GYN into account (weighted Likert scale: 3.31) and whether the current emphasis on promoting ambulatory medicine / general practice in the medical curriculum – which includes a mandatory rotation in the final year (“Praktisches Jahr”) – compromises education in OB/GYN (weighted Likert scale: 3.4) (Table 6).

**Table 6:** Respond regarding the extent to which you agree or disagree with the following statements about general assessments and observations of student teaching in OB/GYN

	completely disagree (1)		disagree (2)		neither agree nor disagree (3)		agree (2)		completely agree (5)		n/a		total	weighted average
	%	*n* =	%	*n* =	%	*n* =	%	*n* =	%	*n* =	%	*n* =	*n* =	
The overall motivation of the students in the subject is present and noticeable	0	0	4	1	23	6	58	15	15	4	0	0	26	3.85
In clinical practice, student teaching is often more of a burden than a form of enrichment	4	1	15	4	42	11	35	9	4	1	0	0	26	3.19
Good and committed teaching is adequately appreciated by the students	0	0	8	2	12	3	54	14	27	7	0	0	26	4
The low interest of male students in further specialization in OB/GYN is problematic	12	3	27	7	35	9	19	5	4	1	4	1	26	2.76
Special teaching offerings for male students could help make the subject more popular in this group	19	5	35	9	27	7	19	5	0	0	0	0	26	2.46
The significant surgical component in obstetrics and gynecology is adequately considered in student teaching	0	0	31	8	15	4	46	12	8	2	0	0	26	3.31
Based on the way that student teaching is currently organized at our faculty, I would have appreciated this kind of teaching during my time as a student	4	1	8	2	31	8	46	12	8	2	4	1	26	3.48
The promotion of outpatient medicine / general medicine with a mandatory rotation during the final year (“Praktisches Jahr”) comes at the expense of education in OB/GYN	8	2	15	4	27	7	23	6	23	6	4	1	26	3.4

## Discussion

In recent years, medical education in Germany has undergone important developments and challenges. The Masterplan for Medical Studies 2020 and the National Competence-Based Learning Objectives for Undergraduate Medical Education (NKLM) have been introduced with the aim of improving – and setting new – quality standards in medical education. By clearly defining learning objectives and competencies, they foster a common understanding among students, educators, and potential employers of the expected skills and knowledge of graduates. Consequently, the NKLM contributes to establishing uniform educational qualities. Moreover, it provides a foundation for designing examinations and assessment methods, making qualifications meaningful and comparable, thereby improving the overall quality of medical education [23]. Despite these clear advantages, the implementation of the NKLM provides significant challenges and additional workload for medical faculties. The challenges arise from the need to realign existing teaching standards with these novel objectives — a task that requires substantial time and effort. According to Nousiainena et al., medical educators face various challenges when implementing a competency-based curriculum, primarily in three areas: restructuring the faculty to accommodate new curricula and assessment methods, revising the teaching and evaluation processes, and altering the educational culture to embrace the competency-based approach [24]. To overcome these challenges, the implementation of the NKLM is an ongoing task and requires continuous evaluations that analyze the practicality and perspectives of implementation of the NKLM and a focus on its further development. To facilitate this, feedback from individual faculties is collectively discussed in committees which include representatives from the faculties, professional societies, student representatives, and other experts. These discussions take place across faculties and have been integrated early into the process of further developing the NKLM [18]. This process offers the faculties not only the opportunity to actively shape the teaching framework themselves but also the chance to anticipate potential challenges that may arise from the licensing regulations for their curriculum and to make necessary adjustments where required. Accordingly, Huber-Lang et al. identified a discrepancy between the competency-oriented frameworks and the "real world" licensing practical-oral medical exam at the beginning of the evaluation and introduction of the NKLM in 2017 [25].

Implementing new teaching structures and methods in line with the NKLM demands considerable time and effort from those in charge. Our study found that a considerable number of teaching coordinators are facing challenges in managing their teaching responsibilities alongside their clinical and research work. This difficulty is exacerbated by a reported lack of dedicated personnel and resources for teaching activities, adding to the complexity of aligning and updating the curriculum. Additionally, there is a significant concern about the transition to competency-based learning objectives as required by new educational standards. While this method is praised for emphasizing practical skills and outcomes, it may inadvertently reduce the time for traditional lectures and classroom teaching. This change risks diminishing the depth of theoretical and fundamental medical knowledge provided to students, a potential side effect that could adversely affect the overall standard of medical education. Kerdijk et. al compared in a Dutch study the learning outcomes of students enrolled in a competency-based curriculum with those in a conventional active-learning curriculum. The findings revealed a significantly poorer performance of students in the competency-based learning curriculum, during the early years of education. This was attributed to a shift in emphasis from the conveyance of theoretical knowledge to the development of practical skills. Furthermore, students in the competency-based curriculum did not demonstrate superior performance in clinical assessments and did not feel better prepared for clinical practice [26].

To address these challenges effectively, it is essential to ensure that those tasked with implementing these changes are well-equipped. This means not only having adequate time but also the necessary qualifications and training to adapt to and embrace these new teaching paradigms. One practical solution could be to reduce the clinical responsibilities of teaching coordinators. This reduction would allow them to dedicate more time and focus on enhancing medical education and its professionalization. Furthermore, encouraging these coordinators to pursue advanced academic degrees in medical education could significantly bolster their ability to manage these changes effectively. An additional postgraduate degree or training has been shown to significantly increase the engagement and performance of educators in the field of medical education [27, 28]. However, the low rate (only 13%) of coordinators currently holding such degrees, as indicated by our study, points to a significant educational and professional development gap that needs to be addressed to ensure the successful implementation of the NKLM.

Although teaching evaluations are regularly conducted in all departments, only 62% of departments distribute (financial) resources based on these evaluations. In order to improve the quality of medical teaching, incentives could serve as a strong motivator for medical faculties. One possible solution to this issue could be to expand financial rewards for good teaching by providing financial support for positive evaluations. This approach could enable additional personnel to be hired and could additionally relieve those who are currently responsible for teaching. In one study that investigated the most potent incentives for boosting the level of motivation and enthusiasm for teaching among basic scientists, residents, and attendings who are actively involved in medical education, medical instructors prioritized monetary incentives and additional time away from their main responsibilities. Career-effective incentives – such as tenure and promotion – were the next respective preferences [29]. Recognizing and rewarding exceptional teaching can foster a culture of excellence and facilitate the continuous improvement of the quality of education. By implementing a system that actively promotes and rewards good teaching, departments can motivate faculty members to strive for better performance, which ultimately benefits not only students, but also the department as a whole. However, the evaluation tools for medical teaching are rather heterogenous in Germany and are not standardized. This situation may result in some uncertainty about the significance of these tools and the compliance of the tools with international quality standards [30].

The results of our study reveal that OB/GYN teaching coordinators acknowledge the importance of practical training in the medical curriculum; however, there are significant shortcomings in both the quality and quantity of the implementation of this practical training. While phantom models are commonly used in OB/GYN teaching, more advanced medical training aids – such as transabdominal or transvaginal ultrasound trainers or hysteroscopy trainers – are not as frequently utilized. The application of such practical models has been proven to enhance the skills of both students and physicians and is highly regarded [31, 32]. Furthermore, most teaching coordinators do not consider performing the gynecological examination to be a fundamental learning objective for students, which may possibly be due to concerns about patient sensitivity or privacy. Although the willingness of patients to consent to gynecological examinations by students largely hinges on the supervising physician's communication skills, one potential area for enhancement could be the inclusion of additional practice sessions with professional patient models. Studies have shown that this approach may improve clinical examination skills in OB/GYN and that it may also increase the frequency of gynecological examinations during clinical clerkships [33].

The teaching coordinators in our study stressed the notion that practical skills that are related to OB/GYN are usually obtained by medical students during their final year or during their medical clerkship. This observation is noteworthy because these modules are not mandatory for students pursuing OB/GYN specialization and must be actively chosen as part of the curriculum. Additionally, learning success is usually more pronounced during direct skill training than during mere participation in a clinical clerkship [34]. Highlighting the critical role of supervision and feedback in medical training, it is essential to acknowledge that these components are key to achieve successful practical learning. Insufficient supervision poses a considerable risk of medical trainees adopting and perpetuating incorrect techniques which could negatively impact patient care and safety. [35]. Feedback, both positive and constructive, is a critical component of effective learning. It helps trainees understand what they are doing right and where they need improvement [36, 37]. In this regard, the instructor does not necessarily have to be a doctor. Good learning outcomes can also be achieved with trained peers which could represent another form of relief for clinically active teachers [38, 39]. Further improvements in practical training are also necessary from the students’ point of view. In another study, 64% of young medical professionals in Germany reported feeling inadequately prepared for the practical requirements of the medical profession [40]. This high percentage of medical professionals feeling underprepared underscores a notable gap between the training provided during medical education and the realities of clinical practice.

Our findings are in line with a previous study that reported that medical students have a general lack of opportunities to perform the gynecological examination [41]. Male medical students, in particular, face a specific challenge in obtaining the necessary practical and hands-on experience in OB/GYN. Our own study on gender differences when it comes to medical students’ choice of specialization at Heidelberg University revealed that male students receive significantly less hands-on training in OB/GYN compared with female students. These findings demonstrate that only a small proportion (22%) of male students gain practical experience in OB/GYN beyond the compulsory bedside teaching during their clinical clerkships, while 62% of male students neither observe nor conduct an examination on their own [42]. Additionally, one publication from the United States highlights the discontent that male students express with their OB/GYN clinical exposure, with gender being cited as a relevant factor in their dissatisfaction [43]. It is important to mention that patient satisfaction with the gynecological examination is not affected by the gender of the examiner [44, 45]. Empathy and effective communication skills play a vital role in this regard [46].

Conducting the gynecological examination is not a relevant goal according to the teaching coordinators. This finding is supported by the findings in another study, in which the main obstacles to obtaining practical experience and skills were found to be medical supervisors who either actively prevented student participation or passively excluded students from opportunities to learn [47]. Studies have shown that positive experiences during medical studies are crucial when it comes to selecting a specialty later on [48–50]. It is important to promote and support students, especially during times of physician shortages. In this regard, mentoring programs can be useful instruments in recruiting graduates by providing guidance during the critical phase of their medical studies. However, our survey revealed that only one-third of OB/GYN departments have established mentoring programs, which indicates that there is significant potential for improvement in this area.

According to the teaching coordinators in our study, urogynecology and reproductive medicine perform the worst when it comes to learning success. With regard to reproductive medicine, this finding is especially interesting because this sub-specialization of OB/GYN is equally as popular among medical students as is gynecological oncology [42]. One potential reason for this discrepancy could be the limited time allocated for teaching these sub-specialties. Given the complex nature of urogynecology and reproductive medicine, which encompasses a range of sensitive and intricate procedures and treatments, the allocated time might be insufficient for students to gain a thorough understanding and proficiency. This finding is particularly significant due to the high prevalence of urogynecological patients and the increasing demand for reproductive medicine over the years [51, 52]. Moreover, the fact that these disciplines are often offered at specialized clinics adds another layer of complexity. This specialization implies that not all medical educators have the expertise or the resources to provide comprehensive training in these areas. The lack of exposure to a diverse range of cases and the absence of specialized mentors might significantly hinder the learning process in urogynecology and reproductive medicine.

In summary, the study provides a comprehensive evaluation of the current state and challenges of medical education in OB/GYN at German universities, particularly in light of recent educational reforms like the Masterplan for Medical Studies 2020 and the NKLM from the viewpoints of the teaching coordinators. While these reforms aim to standardize and improve the quality of medical education, their implementation poses significant challenges, including alignment with existing teaching standards, increased workload for faculty, and the need for continual development and feedback. The study highlights specific concerns in OB/GYN education, such as the struggle of teaching coordinators to balance their clinical, research, and teaching responsibilities, and the potential impact of competency-based learning on the depth of theoretical knowledge. The move towards practical skills and outcomes, while beneficial in certain aspects, may inadvertently reduce the emphasis on essential theoretical knowledge. Furthermore, the study underscores the importance of practical training in medical education, with particular reference to the fields of OB/GYN. The findings suggest significant gaps in both the quality and quantity of practical training, with a notable lack of advanced training aids and insufficient emphasis on fundamental procedures like gynecological examinations. This gap is more pronounced for male students, who receive less hands-on training in OB/GYN.

## Conclusion

The medical education system in Germany is currently in the process of being restructured and faces ongoing challenges. To address these challenges, our study suggests several approaches, for example, reducing the clinical responsibilities of teaching coordinators, providing them with further academic training in medical education, and introducing financial incentives based on teaching evaluations. Additionally, our study calls for more emphasis on practical skills training, improved supervision and feedback during training, and the establishment of mentoring programs to guide students through their medical education. Overall, our study emphasizes the need for continuous evaluation and adaptation of medical education strategies to ensure they meet the evolving needs of students and the medical profession. This includes balancing the focus on practical skills with the preservation of essential theoretical knowledge and addressing the specific challenges faced in various specializations, such as OB/GYN, to enhance the overall quality and effectiveness of medical education in Germany.

## Limitations

With regard to the limitations of this study, the questionnaire from our survey was used here for the first time and had not been previously validated. The cohort of respondents represents a homogeneous group of experienced gynecologists who are primarily responsible for teaching at their universities. Therefore, a possible response bias must be considered. Our evaluation is missing data from 26% of German universities because only 30 of 41 respondents completed the questionnaire. Receiving the missing 26% of responses might have enabled an even more precise analysis and evaluation of current trends in medical teaching. In order to make a final assessment of the state of medical education, teachers’ views should also be compared with students’ experiences in the future. However, we consider it a strength of our analysis to specifically address the teaching coordinators in OB/GYN as they are key stakeholders in the medical education system. Furthermore, it would be intriguing to compare these experiences with the perspectives of patients and healthcare professionals, such as nurses. The impact of education on the daily routines of these groups is also noteworthy. This would contribute to a more comprehensive understanding of the current state of medical education in OB/GYN in Germany. One limitation of our study may also be the time period used for data acquisition (i.e., 19 April to 7 June 2021), during which the COVID-19 pandemic passed its apex in Germany and vaccines became available to the public. By that point, new and digital teaching formats may already have been established. Moreover, due to the high vaccination rate, teaching with a significant amount of patient contact may also have resumed.

### Supplementary Information

**Additional file 1:Supplementary Table 1.** Please provide the first digit of the five-digit postal code (PLZ) of your clinic’s address.

## Data Availability

All (raw) data and material are available upon reasonable request. Please contact the corresponding author (Maximilian Riedel).

## Abbreviations

OB/GYN: Obstetrics and gynecology
PJ: Praktisches Jahr
NKLM: National Competency-Based Learning Objectives for Undergraduate Medical Education
OSCE: Objective Structured Clinical Examination
Mini-CEX: Mini-Clinical Evaluation Exercises
POL: Problem-based learning

## References

[1] Nikendei C, Weyrich P, Jünger J, Schrauth M (2009). Medical education in Germany. Med Teach.

[2] Nikendei C, Krautter M, Celebi N, Obertacke U, Jünger J (2012). Final Year Medical Education in Germany. Z Evid Fortbild Qual Gesundhwes.

[3] Riedel M, Amann N, Recker F, Hennigs A, Heublein S, Meyer B (2022). The COVID-19 pandemic and its impact on medical teaching in obstetrics and gynecology-A nationwide expert survey among teaching coordinators at German university hospitals. PLoS ONE.

[4] Jünger J (2018). Competence-based assessment in the national licensing examination in Germany. Bundesgesundheitsblatt Gesundheitsforschung Gesundheitsschutz.

[5] The Federal Joint Committee. Available from: https://www.g-ba.de/english/

[6] Schiekirka-Schwake S, Dreiling K, Pyka K, Anders S, von Steinbüchel N, Raupach T (2018). Improving evaluation at two medical schools. Clin Teach.

[7] Junges Forum in der DGGG. 2023. Available from: https://www.dggg.de/weiterbildung-nachwuchs/junges-forum

[8] Speidel R, Schneider A, Körner J, Grab-Kroll C, Öchsner W (2021). Did video kill the XR star? Digital trends in medical education before and after the COVID-19 outbreak from the perspective of students and lecturers from the faculty of medicine at the University of Ulm. GMS J Med Educ..

[9] Loda T, Löffler T, Erschens R, Zipfel S, Herrmann-Werner A (2020). Medical education in times of COVID-19: German students’ expectations - A cross-sectional study. PLoS ONE.

[10] Holzmann-Littig C, Zerban NL, Storm C, Ulhaas L, Pfeiffer M, Kotz A (2022). One academic year under COVID-19 conditions: two multicenter cross-sectional evaluation studies among medical students in Bavarian medical schools, Germany students’ needs, difficulties, and concerns about digital teaching and learning. BMC Med Educ.

[11] Kaul V, Gallo de Moraes A, Khateeb D, Greenstein Y, Winter G, Chae J (2021). Medical Education During the COVID-19 Pandemic. Chest.

[12] Torda A (2020). How COVID-19 has pushed us into a medical education revolution. Intern Med J.

[13] Sandars J, Patel R (2020). The challenge of online learning for medical education during the COVID-19 pandemic. Int J Med Educ.

[14] Fritze O, Griewatz J, Narciß E, Shiozawa T, Wosnik A, Zipfel S (2017). How much GK is in the NKLM? A comparison between the catalogues of exam-relevant topics (GK) and the German National Competence-based Learning Objectives Catalogue for Undergraduate Medical Education (NKLM). GMS J Med Educ..

[15] Blaum WE, Dannenberg KA, Friedrich T, Jarczewski A, Reinsch AK, Ahlers O. Der praktische Nutzen des Konsensusstatements “praktische Fertigkeiten im Medizinstudium” – eine Validierungsstudie. GMS Zeitschrift für Medizinische Ausbildung; 29(4):Doc58; ISSN 1860–3572. 2012 [cited 2023 Feb 20]; Available from: http://www.egms.de/en/journals/zma/2012-29/zma000828.shtml 10.3205/zma000828 PMC3420120 22916084

[16] Koch K, Hirt B, Shiozawa-Bayer T, Königsrainer A, Fusco S, Wichmann D. Development of an interactive elective “altered anatomy” for students as part of the Z-curriculum according to the NKLM 2.0. 2023 [cited 2023 Dec 12]; Available from: https://www.egms.de/en/journals/zma/2023-40/zma001625.shtml 10.3205/zma001625 PMC10407590 37560042

[17] Plange N, Feltgen N, Arbeitsgemeinschaft Lehre (2023). [The “Nationaler Kompetenzbasierter Lernzielkatalog Medizin NKLM 2.0”-Implications for medical education in ophthalmology]. Ophthalmologie.

[18] Evaluation und Weiterentwicklung des NKLM. 2022. Available from: https://nklm.de/zend/videos/FAQ_zu_NKLM-Evaluation_und_-Weiterentwicklung.pdf

[19] Frank JR, Snell LS, Cate OT, Holmboe ES, Carraccio C, Swing SR (2010). Competency-based medical education: theory to practice. Med Teach.

[20] Katoue MG, Schwinghammer TL (2020). Competency-based education in pharmacy: A review of its development, applications, and challenges. J Eval Clin Pract.

[21] Chimea TL, Kanji Z, Schmitz S (2020). Assessment of clinical competence in competency-based education. Can J Dent Hyg.

[22] Hammad N, Ndlovu N, Carson LM, Ramogola-Masire D, Mallick I, Berry S (2023). Competency-Based Workforce Development and Education in Global Oncology. Curr Oncol.

[23] Söhnel A, Frankenberger R, Kandsperger L, Wissing F (2023). NKLZ 2.0: Die Weiterentwicklung des Nationalen Kompetenzbasierten Lernzielkatalogs Zahnmedizin als Basis für die Ausgestaltung der neuen Approbationsordnung. Bundesgesundheitsbl..

[24] Nousiainen MT, Caverzagie KJ, Ferguson PC, Frank JR (2017). on behalf of the ICBME Collaborators. Implementing competency-based medical education: What changes in curricular structure and processes are needed? Medical Teacher..

[25] Huber-Lang M, Palmer A, Grab C, Boeckers A, Boeckers TM, Oechsner W (2017). Visions and reality: the idea of competence-oriented assessment for German medical students is not yet realised in licensing examinations. GMS J Med Educ..

[26] Kerdijk W, Snoek JW, van Hell EA, Cohen-Schotanus J (2013). The effect of implementing undergraduate competency-based medical education on students’ knowledge acquisition, clinical performance and perceived preparedness for practice: a comparative study. BMC Med Educ.

[27] Sethi A, Schofield S, McAleer S, Ajjawi R (2018). The influence of postgraduate qualifications on educational identity formation of healthcare professionals. Adv in Health Sci Educ.

[28] Armstrong EG, Doyle J, Bennett NL (2003). Transformative Professional Development of Physicians as Educators: Assessment of a Model. Acad Med.

[29] Hofer M, Pieper M, Sadlo M, Reipen J, Heussen N (2008). Performance-related middle management in medical teaching. Attractiveness of incentive tools from the perspective of the teachers. Dtsch Med Wochenschr.

[30] Schiekirka-Schwake S, Barth J, Pfeilschifter J, Hickel R, Raupach T, Herrmann-Lingen C. National survey of evaluation practices and performance-guided resource allocation at German medical schools. GMS German Medical Science; 17:Doc04. 2019 [cited 2023 Mar 6]; Available from: http://www.egms.de/en/journals/gms/2019-17/000270.shtml 10.3205/000270 PMC6533543 31148955

[31] Le C, Lewis J, Steinmetz P, Dyachenko A, Oleskevich S (2019). The Use of Ultrasound Simulators to Strengthen Scanning Skills in Medical Students: A Randomized Controlled Trial. J of Ultrasound Medicine.

[32] Taksøe-Vester C, Dyre L, Schroll J, Tabor A, Tolsgaard M (2021). Simulation-Based Ultrasound Training in Obstetrics and Gynecology: A Systematic Review and Meta-Analysis. Ultraschall Med.

[33] Siwe K, Wijma K, Stjernquist M, Wijma B (2007). Medical students learning the pelvic examination: Comparison of outcome in terms of skills between a professional patient and a clinical patient model. Patient Educ Couns.

[34] Vogel D, Harendza S. Basic practical skills teaching and learning in undergraduate medical education – a review on methodological evidence. GMS Journal for Medical Education; 33(4):Doc64. 2016 Aug 15 [cited 2023 Mar 13]; Available from: http://www.egms.de/en/journals/zma/2016-33/zma001063.shtml 10.3205/zma001063 PMC5003143 27579364

[35] Fischer T, Chenot JF, Simmenroth-Nayda A, Heinemann S, Kochen MM, Himmel W (2007). Learning core clinical skills—a survey at 3 time points during medical education. Med Teach.

[36] Weissenbacher A, Bolz R, Zimmermann A, Donaubauer B, Stehr SN, Hempel G (2021). Mentoring und arbeitsplatzbasierte Prüfungen im Praktischen Jahr: Ein effektiver Weg zur Steigerung von Zufriedenheit und Kompetenz?. Anaesthesist.

[37] Lauterjung ML, Ehlers C, Guntinas-Lichius O (2021). PJplus - a project improving practical training during the final year of medical education. Z Evid Fortbild Qual Gesundhwes.

[38] Ruiz JG, Mintzer MJ, Leipzig RM (2006). The Impact of E-Learning in Medical Education: Academic Medicine.

[39] Weyrich P, Celebi N, Schrauth M, Möltner A, Lammerding-Köppel M, Nikendei C (2009). Peer-assisted versus faculty staff-led skills laboratory training: a randomised controlled trial. Med Educ.

[40] Dannenberg KA, Stroben F, Schröder T, Thomas A, Hautz WE. The future of practical skills in undergraduate medical education – an explorative Delphi-Study. GMS Journal for Medical Education; 33(4):Doc62. 2016 Aug 15 [cited 2023 Mar 13]; Available from: http://www.egms.de/en/journals/zma/2016-33/zma001061.shtml 10.3205/zma001061 PMC5003134 27579362

[41] Danielsson J, Hadding C, Fahlström M, Ottander U, Lindquist D (2021). Medical students’ experiences in learning to perform pelvic examinations: a mixed-methods study. Int J Med Educ.

[42] Riedel M, Hennigs A, Dobberkau AM, Riedel C, Bugaj TJ, Nikendei C, et al. The role of gender-specific factors in the choice of specialty training in obstetrics and gynecology: results from a survey among medical students in Germany. Arch Gynecol Obstet. 2021 Sep 22 [cited 2021 Oct 17]; Available from: https://link.springer.com/10.1007/s00404-021-06232-2 10.1007/s00404-021-06232-2 PMC8782790 34550446

[43] Wallbridge T, Holden A, Picton A, Gupta J (2018). Does medical students’ gender affect their clinical learning of gynaecological examination? A retrospective cohort study. Postgrad Med J.

[44] Childs AJ, Friedman WH, Schwartz MP, Johnson M, Royek AB (2005). Female Patientsʼ Sex Preferences in Selection of Gynecologists and Surgeons: Southern Medical Journal.

[45] Johnson AM, Schnatz PF, Kelsey AM, Ohannessian CM (2005). Do women prefer care from female or male obstetrician-gynecologists? A study of patient gender preference. J Am Osteopath Assoc.

[46] Plunkett BA, Kohli P, Milad MP (2002). The importance of physician gender in the selection of an obstetrician or a gynecologist. Am J Obstet Gynecol.

[47] van den Einden LCG, te Kolste MGJ, Lagro-Janssen ALM, Dukel L (2014). Medical students’ perceptions of the physician’s role in not allowing them to perform gynecological examinations. Acad Med.

[48] Mihalynuk T, Leung G, Fraser J, Bates J, Snadden D (2006). Free choice and career choice: Clerkship electives in medical education. Med Educ.

[49] Riedel F, Riedel M, Freis A, Heil J, Golatta M, Schuetz F (2019). Exam preparatory course for the 2nd part of the German medical examination in obstetrics and gynecology – a potential tool for the recruitment of new residents during the occupational decision process before the practical year?. BMC Med Educ.

[50] Dornan T, Littlewood S, Margolis SA, Scherpbier A, Spencer J, Ypinazar V (2006). How can experience in clinical and community settings contribute to early medical education?. A BEME systematic review Medical Teacher.

[51] Gliozheni O, Hambartsoumian E, Strohmer H, Petrovskaya E, Tishkevich O, European IVF Monitoring Consortium (EIM), for the European Society of Human Reproduction and Embryology (ESHRE) (2022). ART in Europe, 2018: results generated from European registries by ESHRE. Human Reproduction Open.

[52] Mascarenhas MN, Flaxman SR, Boerma T, Vanderpoel S, Stevens GA (2012). National, Regional, and Global Trends in Infertility Prevalence Since 1990: A Systematic Analysis of 277 Health Surveys. PLoS Med..

