# Supplementary material for: Exploring the current state of clinical and practical teaching in obstetrics and gynecology in the era of competency-based education: a nationwide survey among German teaching coordinators
Source: BMC Med Educ. 2024 Feb 21;24:165. doi: 10.1186/s12909-024-05138-2 (PMC10880315; doi:10.1186/s12909-024-05138-2)
Supplement: Supplementary file 1 — Additional file 1:Supplementary Table 1. Please provide the first digit of the five-digit postal code (PLZ) of your clinic’s address. [file 12909_2024_5138_MOESM1_ESM.docx]

**Supplementary Table 1**

Please provide the first digit of the five-digit postal code (PLZ) of your clinic’s address.

| **postal code (first digit)** | **response** | |
| --- | --- | --- |
|  | **%** | **n=** |
| **0** | 7 | 2 |
| **1** | 3 | 1 |
| **2** | 7 | 2 |
| **3** | 13 | 4 |
| **4** | 17 | 5 |
| **5** | 10 | 3 |
| **6** | 10 | 3 |
| **7** | 10 | 3 |
| **8** | 13 | 4 |
| **9** | 10 | 3 |
